# Differences in isometric strength and sprinting speed of academy soccer players: a special interest in transition between the age groups and national team selected players

**DOI:** 10.3389/fspor.2025.1630522

**Published:** 2025-07-17

**Authors:** Martin Mikulič, Jozef Cholp, Nikolas Nagy, Filip Skala

**Affiliations:** ^1^ Department of Sports Games, Faculty of Physical Education and Sport, Comenius University in Bratislava, Bratislava, Slovakia; ^2^ Department of Biological and Medical Sciences, Faculty of Physical Education and Sport, Comenius University in Bratislava, Bratislava, Slovakia; ^3^ Department of Strength and Conditioning, Football Club DAC 1904 Dunajská Streda, Dunajská Streda, Slovakia

**Keywords:** talent identification, neuromuscular performance, maturity status, agility, youth soccer

## Abstract

Players in European soccer academies frequently advance from the lower to the higher age group. Underdevelopment of their strength and speed capacities increases the risk of injury. On the one hand, this study compared the isometric strength and speed performance of elite youth soccer players regarding age groups. Further, it aimed to recognize differences in relative isometric strength between the national team selected and non-selected academy players. Eighty-three academy players from five age groups of single academy were compared in isometric peak force production in a bilateral knee flexion test (ISO 30°), abduction and adduction tests (ABD and ADD 60°). Their sprinting speed was evaluated by 10 and 30 meter sprint tests, and the 505 change of direction test (COD 180°). Significant age group effects were discovered for absolute isometric strength (*p* = < 0.001; *η*^2^ = 0.40–0.43) but relative strength differed only between U15 and U19 in ISO 30° (*p* = 0.04; ES = 1.04). The U14 players were slower than all groups (ES = 0.95–3.68) excluding the U15. These players were slower than U16, U17, and U19 (ES = 1.07–2.37), while U17 overpassed U19 in 180° COD (*p* = 0.02; ES = 0.22). Consequently, sprinting speed demands are of special interest in the transition of players from the U15 to the U16 age group. The national team players were not relatively stronger in ISO 30° (–0.08 *N*/kg, *p* = 0.70; ES = 0.10), ABD 60° (–0.14 *N*/kg, *p* = 0.59; ES = 0.13), and ADD 60° (–0.33 *N*/kg, *p* = 0.22; ES = 0.31) compared to their academy peers This suggests that maximal relative isometric strength is not of special interest for the selection of academy soccer players to the national team squad.

## 1 Introduction

The performance of elite soccer players is a complex phenomenon encompassing diverse aspects such as strength, speed, agility, endurance, and coordination. Each of these factors contributes to their overall success on the field (1, 2). Physical preparation is crucial for optimal soccer performance not only for professionals but also for young players. Besides the increased amount of high-intensity actions occurring at the elite level of soccer (3), the similar trend is visible in the matches of youth teams (4). Therefore, young players must gradually develop their physical aspects of performance to cope with these increased game demands (5).

The assessment of physical performance is well structured in soccer academies worldwide. It aims to monitor the physical development of players and to optimize their training programs (6). It is established that muscular strength enhances general athletic abilities such as jumping, sprinting, and change of direction speed. Moreover, it contributes to the decreased risk of injury (7). Muscular strength can be assessed using concentric, eccentric, and isometric muscle contractions. Sport science research has increasingly adopted topics investigating the isometric contraction, a type of muscle contraction in which the muscle-tendon unit maintains a constant length [see e.g., (8–10)]. The isometric strength assessment demands neuromuscular functions by producing the maximal isometric voluntary contraction of a specific muscle group (11). This testing is easier and safer for the setup compared to the traditional one-repetition maximum method. For example, force produced during the isometric mid-thigh pull exercise demonstrates high reliability among youth athletes (12), and it well correlates with one-repetition maximal testing (13). Furthermore, isometric muscle contraction results in lower levels of fatigue compared to dynamic contractions (14). These aspects are very important to consider for the assessment procedures in youth athletes. Therefore, isometric testing may serve as a useful testing method for soccer players in the development phase.

Maximal isometric strength of posterior kinetic chain muscles such as glutes, hamstrings, and trunk extensors highly correlates with linear sprinting speed (15) and change of direction speed (16) in youth soccer players. It is generally assumed that stronger players elicit better results in speed testing. To our knowledge, there are limited number of studies describing the normative data and comparing the age group differences for isometric strength parameters among elite youth soccer players. Keytsman et al. (10), measured isometric strength of knee flexors in isolated seated position in 45° angle in knee joint. Age had no significant influence on the strength profile of Belgian youth soccer players. Ishøi et al. (17), used handheld dynamometer to measure isometric strength of hamstring muscles in prone position with 15° knee flexion angle. These data showed progressive increment of isometric strength from U13 to U19 Danish academy players. Another study also used the isolated assessment of knee flexors strength (18) aiming to bring normative data for relative strength in 18–24 years old soccer players. However, there are missing links in age related differences of pubescent and adolescent soccer players regarding their isometric strength parameters. Simultaneously, this issue is evident for their abduction and adduction force production. Recent study has shown that adduction and abduction strength increases with age from U14 to U19 age group (19). Understanding the normative values and the variations in isometric strength parameters across different age groups may emphasize the significance of muscular strength development throughout the transition from lower to higher age groups.

Players in European soccer academies are categorized into age groups based on their chronological age. However, players frequently advance from the lower to the higher age group in favor of their technical and tactical skills. Therefore, the neuromuscular development of these athletes must not be neglected, as later maturation and advanced chronological age are potential risk factors for injury in elite male youth soccer players (20). Significant variations in the physical and psychosocial development of athletes exist within age groups (21). Biological maturity correlates positively with match locomotion and overall physical capacity, with more mature players exhibiting better sprinting performance (22). For example, mature players cover more high-intensity distance, accelerate and decelerate more often during matches. In addition, they are capable to achieve higher maximal speed compared to their peers (23). Maturation is the most prevalent in age categories U15 and U16. In this period, there is a huge variance in the level of peak growth state. This puts early maturers into clear physical advantage against their late maturing counterparts (24).

The levels of maximal strength and sprinting speed are important in the talent identification procedures in soccer. Players selected to soccer academies achieve higher levels of isometric strength, speed, and agility compared to peers who are not selected (25). It is suggested that elite youth soccer players have greater maximal voluntary force capabilities than their non-elite counterparts (26). Even though the selection of players is often influenced by biological maturation (27, 28), it is not known whether relative isometric strength influence their selection to the national teams.

Building upon these insights, this study aims to compare the isometric strength, sprinting speed, and change of direction speed of elite youth soccer players, with a focus on differentiating the national team selected players from their non-selected counterparts. Examination and comparison of these attributes would point to the specific age groups where the levels of strength and speed may be crucial to consider for the transitioning of players from lower to higher age group or for the selection of players into the academy squad. This research seeks recommendations for testing and training based on the developmental requirements of athletes in elite soccer academies.

## 2 Materials and methods

### 2.1 Subjects

Eighty-three elite youth soccer players from the same local first-tier academy were voluntarily recruited to participate in our study. These players were available for unrestricted training and competitive games. Testing was determined by academy performance and medical staff (i.e., club physiotherapists, strength and conditioning coaches). Subjects had a minimum of 5 years of experience in regular soccer training (4× per week; 80–100-min) with an official eleven-a-side played match during the weekend (2 × 40-min for U14, U15, and U16; 2 × 45-min for U17 and U19), and with a minimum of 1 year of experience in structured strength training within the academy (1–2× per week; 30–45-min.). Subjects were assigned to their age group based on their chronological age (e.g., players born in the period between 01/01/2011 and 31/12/2011 were considered U14). Players selected to the national team in the period of last six months prior to the testing were considered as national team selected players. Descriptive statistics for the subjects are provided in Table 1.

**Table 1:** Anthropometric data of academy soccer players (mean ± SD).

Group	*n*	Age (y)	Height (cm)	Body mass (kg)
U14	15	13.1 ± 1.2	163.2 ± 8.9	48.2 ± 8.7
U15	12	14.0 ± 0.5	172.5 ± 7.5	62.3 ± 11.5
U16	20	15.1 ± 0.3	176.9 ± 7.3	65.8 ± 6.6
U17	14	16.1 ± 0.3	179.1 ± 5.0	68.8 ± 9.4
U19	22	17.5 ± 0.7	178.0 ± 5.9	70.4 ± 5.9

This study was conducted as part of the regular physical performance testing of players within the soccer academy. Players who suffered any forms of musculoskeletal injuries or health disorders prior to the testing were excluded from this study. Parental consent and player assent were obtained prior to testing. This research was conducted in accordance with the Declaration of Helsinki and approved by the local ethics committee (no. 17/2024, date: 10/01/2025).

### 2.2 Experimental approach to the problem

A cross-sectional design examining players of five age groups was used for the purpose of this study. Testing was executed in the pre-season period of the season 2024/2025 in Slovakia. It was carried out over a one-day period. The days and times of testing were different for the U14 and U15 groups (Tuesday from 3 to 6 pm) and the U16 to U19 groups (Wednesday from 8 to 12 am). The tests were completed after a rest day to limit any potential fatigue effects. Subjects were asked to avoid any physical activity for 24 hours prior to the testing and to sleep at least 8 hours a night. Firstly, they underwent the anthropometric measurements where body mass and height were assessed by the academy medical staff. After the standardized 15-min warm-up, which consisted of mobility and activation exercises done by strength and conditioning coaches, players performed three repetitions of three isometric tests. All players were familiar with the tests, as they were part of their academy testing routine. These tests were undertaken at the academy gym. After this part of the session, participants went outdoors to perform another specific 10-min warm-up that consisted of jogging and athletic drills. Their linear and change of direction speed was then tested on the regular artificial grass field. They used soccer-specific footwear in this part of the testing. A study protocol was performed in similar environmental conditions for both testing days (i.e., temperatures ranging from 25°C to 30°C with wind absence).

### 2.3 Procedures

#### 2.3.1 Isometric strength of knee flexors

The maximal isometric strength of the knee flexor muscles (ISO 30°) was evaluated using the NordBord device (Vald Performance, Newstead, Australia), with data sampled at a frequency of 50 Hz. This device provided valid and reliable measurements of eccentric and isometric strength [CV = 9.52%; ICC = 0.73 (29);]. This test simulates the angle of the knee joint during the “late swing phase” of the running gait cycle. Participants began in a kneeling position with hooks placed around the upper parts of the ankles (lateral malleolus). Their knees were bent at an angle of 30 degrees, with their forearms on the ground. They were instructed to maintain their legs in the same position while slightly maintaining contact with the hooks. They subsequently applied force to the hooks with both legs for five seconds at maximum intensity and then relaxed. A single set of three bilateral repetitions was executed, with a 10-second rest interval. The maximal isometric force produced for each leg was determined in Newtons and averaged from the highest of three repetitions. Relative values were calculated as the maximal force output generated during exercise divided by the body mass of each subject (*N*/kg). An average force of right and left leg was considered for the analysis.

#### 2.3.2 Isometric strength of hip abductors and adductors

The maximal isometric strength of abduction (ABD 60°) and adduction (ADD 60°) was evaluated using the ForceFrame device (Vald Performance, Newstead, Australia). The device provides a reliable evaluation of maximal hip strength in young soccer players [ICC = 0.77–0.95; (30)]. Acceptable test-retest reliability for the assessment of maximal force in ABD and ADD 60° tests was demonstrated elsewhere [CV% = 5.0–5.7; (31)]. The participants were positioned supine with 60 degrees of hip flexion. The femoral and tibial condyles were positioned with the inner force pads to assess hip adduction, whilst the lateral femoral condyle and fibular head were aligned with the outer force pads to assess hip abduction strength. Participants were instructed to maintain contact with the floor and refrain from elevating their legs, pelvis, or head during the 5-second maximal voluntary isometric contractions for adduction and abduction. A single set of three bilateral repetitions was executed, with a 10-second rest interval among the contractions. The maximal force outputs during a 5 s pushing intervals were collected as results. Each repetition was separated by a 10-second interval (32). The maximal isometric force output was measured for each leg (in Newtons) and averaged from the highest of three repetitions for statistical analysis. Relative values were calculated according to the previous description. An average of the production of force in the right and left leg was considered for the analysis.

#### 2.3.3 Sprinting speed test

The linear sprinting speed was assessed by a single-beam timing system (Witty Gate, Microgate, Bolzano, Italy). Timing gates were positioned at 10- and 30-meters distances from the starting line. The initial gate was placed at the starting line. The dimensions of the gates were 1.5 m in width and 90 cm in height. Subjects began from a standing position, 30 cm behind the starting line. They were asked to accelerate with full exertion. In total, three trials of 30-meter sprints, including 10-meter split times, were conducted. Three-minute rest periods were incorporated between trials. The lowest times from the repetitions were used for further analysis.

#### 2.3.4 Change of direction speed test

Change of direction speed of players was assessed by the 505 Agility Test (180° COD) (33). A single-beam timing system Witty Gate (Microgate, Bolzano, Italy) was used. Subjects began from a standing position 10 m away from the timing gate. They were instructed to sprint through the gate, execute a 180° turn on the white line located 5 m away from the gate, and then reaccelerate back to the gate. Two trials for each foot placement were performed. The lowest time from all the repetitions was used for the analysis.

### 2.4 Statistical analysis

Data are presented as means ± standard deviations (SD) for each age group. A Shapiro–Wilk test was used to evaluate the normal distribution of the data. Homogeneity of variance was evaluated by the Levene's test. To analyze the differences between age groups, a one-way analysis of variance (ANOVA) with Tukey's *post hoc* test was conducted. Independent t-tests were performed to compare age groups in absolute strength, speed, and relative strength between selected and non-selected national team players. The significance level was set at *α* = 0.05. The effect size (ES) for the ANOVA analysis was determined by calculating eta-square (*η*^2^). The range of ES was determined as follows: *η*^2^ < 0.01: negligible; 0.01 ≤ *η*^2^ < 0.06: small; 0.06 ≤ *η*^2^ < 0.14: medium; *η*^2^ ≥ 0.14: large. The *post hoc* statistical power for the comparison of selected and non-selected national team players was calculated with G*Power (Version 3.1.9.6, Institut für Experimentelle Psychologie, Düsseldorf, Germany). The power for the number of subjects within the study sample was 1−β = 0.613 with α = 0.05. To compensate for the lower sample size power, the Cohen's d effect sizes with 95% confidence intervals were calculated. Cohen's d was regarded as a trivial (d < 0.2), small (0.2 ≤ d < 0.5), medium (0.5 ≤ d < 0.8), or large (d ≥ 0.8) (34). Statistical analysis was performed by SPSS software 28.0.1.1 (SPSS Inc., Chicago, IL, US), while for the graphical analysis GraphPad Prism 10.3.1 (GraphPad Software, San Diego, CA, USA) was used.

## 3 Results

Descriptive statistics for absolute and relative maximal isometric strength of each age group are shown in Table 2. Sprinting speed variables of these age groups are described in Table 3.

**Table 2:** Absolute (*N*) and relative (*N*/kg) maximal isometric strength of academy players by age groups.

Test	Age group	M	SD (±)	F	*p* (*η*^2^)	Post-hoc test (*p*, ES)
ISO 30° [*N*]	U14	229	50.4	13	< 0.001 (0.40)	U14 < U16 (*p* < 0.001, 1.61) U14 < U17 (*p* < 0.001, 2.12) U14 < U19 (*p* < 0.001, 2.87) U15 < U17 (*p* < 0.001, 0.87) U15 < U19 (*p* < 0.001, 1.23)
	U15	276	99.4			
	U16	325	67.1			
	U17	348	61.1			
	U19	373	49.9			
ISO 30° [*N*/kg]	U14	4.83	1.04	2.27	0.069 (0.10)	U15 < U19 (*p* < 0.043, 0.94)
	U15	4.45	1.10			
	U16	4.88	0.70			
	U17	5.08	0.74			
	U19	5.31	0.67			
ABD 60° [*N*]	U14	229	63.4	13.5	< 0.001 (0.41)	U14 < U16 (*p* < 0.001, 1.55) U14 < U17 (*p* < 0.001, 2.40) U14 < U19 (*p* < 0.001, 2.02) U15 < U17 (*p* < 0.001, 1.26) U15 < U19 (*p* < 0.001, 1.05)
	U15	297	70.2			
	U16	331	67.9			
	U17	381	63.3			
	U19	366	61.0			
ABD 60° [*N*/kg]	U14	4.98	1.76	0.62	0.650 (0.03)	
	U15	5.00	1.60			
	U16	4.99	0.90			
	U17	5.55	0.68			
	U19	5.20	0.86			
ADD 60° [N]	U14	265	44.1	14.8	< 0.001 (0.43)	U14 < U15 (*p* < 0.001, 1.28) U14 < U16 (*p* < 0.001, 2.28) U14 < U17 (*p* < 0.001, 2.42) U14 < U19 (*p* < 0.001, 2.56)
	U15	341	71.3			
	U16	371	48.7			
	U17	404	68.4			
	U19	394	56.0			
ADD 60° [*N*/kg]	U14	5.71	1.47	0.15	0.962 (0.01)	
	U15	5.70	1.54			
	U16	5.63	0.83			
	U17	5.89	0.73			
	U19	5.62	0.88			

M, mean value; SD, standard deviation; F, f-value for one-way ANOVA; *p*, statistical significance; η^2^, eta squared effect size; ES, effect size; ISO 30°, isometric strength test of the knee flexors; ABD 60°, isometric strength test of the hip abductors; ADD 60°, isometric strength test of the hip adductors.

**Table 3:** Linear and change of direction speed performance of academy soccer players by age groups.

Test	Age group	M	SD (±)	F	*p* (η^2^)	Post-hoc test (*p*, ES)
10 m sprint [s]	U14	1.95	0.07	22	<0.001 (0.53)	U14 < U16 (*p* < 0.001, 2.60) U14 < U17 (*p* < 0.001, 1.96) U14 < U19 (*p* < 0.001, 2.94) U15 < U16 (*p* < 0.001, 1.56) U15 < U17 (*p* < 0.001, 1.07) U15 < U19 (*p* < 0.001, 1.82)
	U15	1.89	0.10			
	U16	1.75	0.08			
	U17	1.79	0.09			
	U19	1.73	0.08			
30 m sprint [s]	U14	4.78	0.16	28	<0.001 (0.59)	U14 < U16 (*p* < 0.001, 3.48) U14 < U17 (*p* < 0.001, 2.54) U14 < U19 (*p* < 0.001, 3.59) U15 < U16 (*p* < 0.001, 1.67) U15 < U17 (*p* < 0.001, 1.22) U15 < U19 (*p* < 0.001, 1.33)
	U15	4.62	0.31			
	U16	4.20	0.17			
	U17	4.29	0.22			
	U19	4.20	0.16			
COD 180° [s]	U14	2.54	0.07	19.7	<0.001 (0.50)	U14 < U16 (*p* < 0.001, 1.95) U14 < U17 (*p* < 0.001, 3.68) U14 < U19 (*p* < 0.001, 2.00) U15 < U16 (*p* < 0.001, 1.39) U15 < U17 (*p* < 0.001, 2.37) U15 < U19 (*p* < 0.001, 1.28)
	U15	2.52	0.11			
	U16	2.37	0.11			
	U17	2.31	0.06			
	U19	2.40	0.07			

M, mean value; SD, standard deviation; F, f-value for one-way ANOVA; *p*, statistical significance; η^2^, eta squared effect size; ES, effect size; ISO 30°, isometric strength test of the knee flexors; ABD 60°, isometric strength test of the hip abductors; ADD 60°, isometric strength test of the hip adductors.

### 3.1 Comparison of isometric strength among age groups

**Figure 1:**
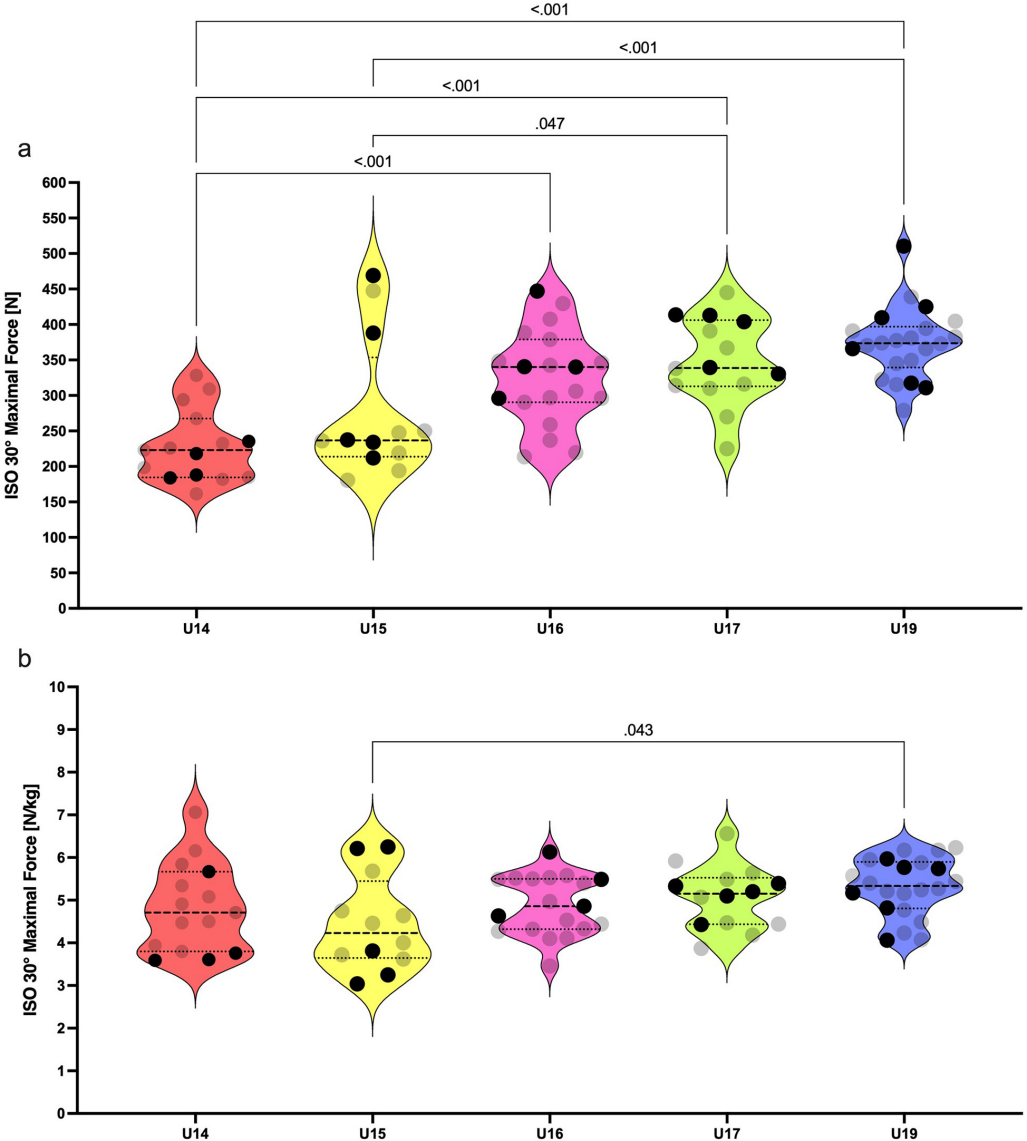
Violin plots for absolute **(a)** and relative **(b)** isometric strength of knee flexors among U14 to U19 players. Black dots represent the national team selected players. Only significant differences are highlighted by pairwise comparison (*p* < .05).

Absolute isometric strength significantly differed among groups in ISO 30° [F _(4, 77)_ = 13.0; *p* = < 0.001; *η*^2^ = 0.40; Figure 1a]. The U14 group produced significantly less force compared to U16, U17, and U19 (ES = 1.61, 2.12, 2.87; respectively). U15 produced less absolute force compared to U17 and U19 (ES = 0.87 and 1.23). Age group had a small effect on the relative force production in ISO 30° [F _(4, 77)_ = 2.27; *p* = 0.069; *η*^2^ = 0.10; Figure 1b] Significant differences were observed only between U15 and U19 (ES = 0.94).

**Figure 2:**
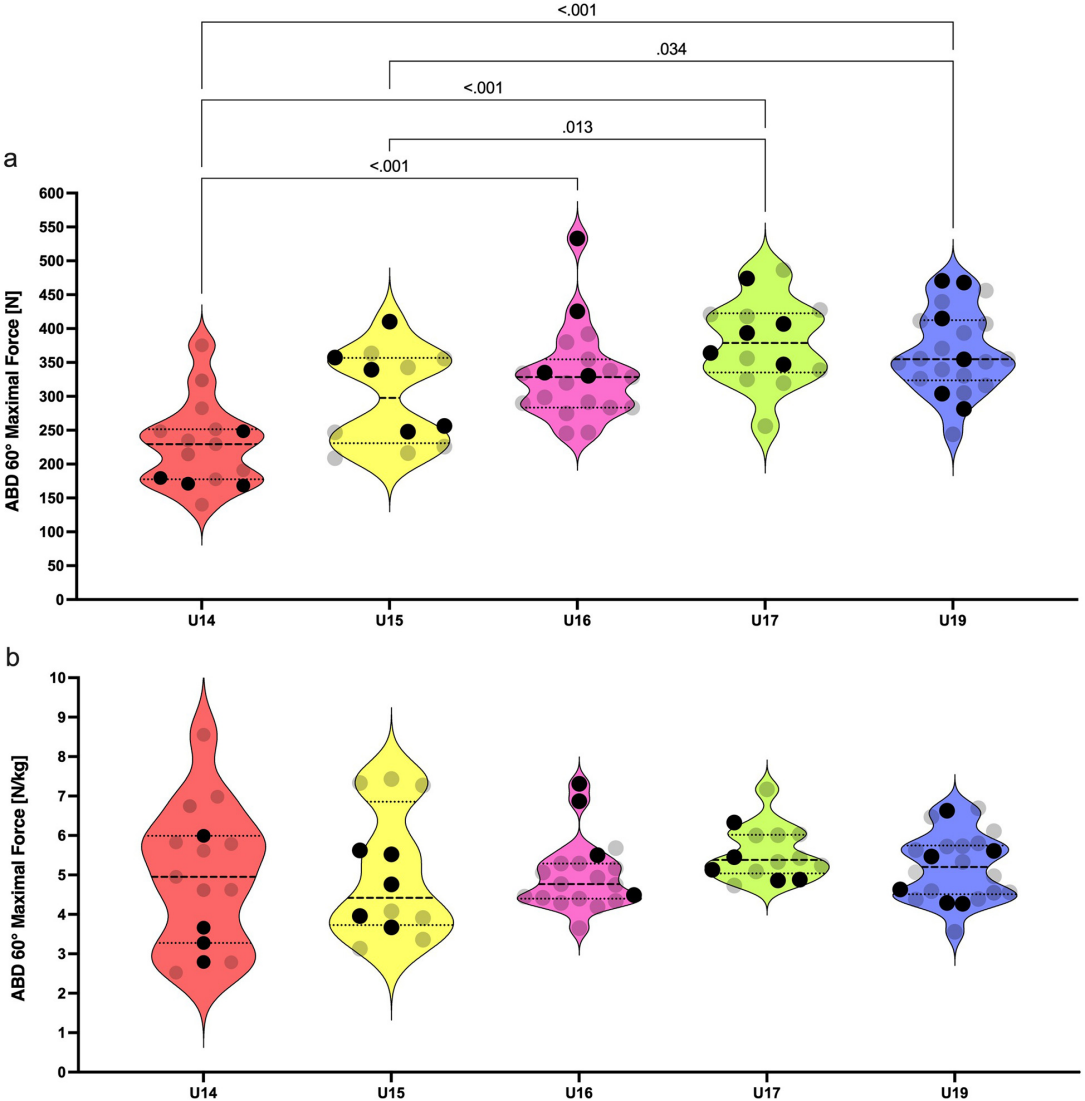
Violin plots for absolute **(a)** and relative **(b)** isometric strength of hip abductors among U14–U19 players. Black dots represent the national team selected players. Only significant differences are highlighted by pairwise comparison (*p* < .05).

Absolute force produced in ABD 60° was influenced by the age group [F (4, 77) = 13.5; *p* = < 0.001; *η*2 = 0.41; Figure 2a] without effect on the relative force in ABD 60° [F (4, 77) = 0.62; *p* = 0.650; *η*2 = 0.03; Figure 2b]. The U14 group produced significantly less force in comparison to U16, U17, and U19 (ES = 1.55, 2.40, 2.02; respectively). U15 produced less force compared to U17 and U19 (ES = 1.26 and 1.06).

**Figure 3:**
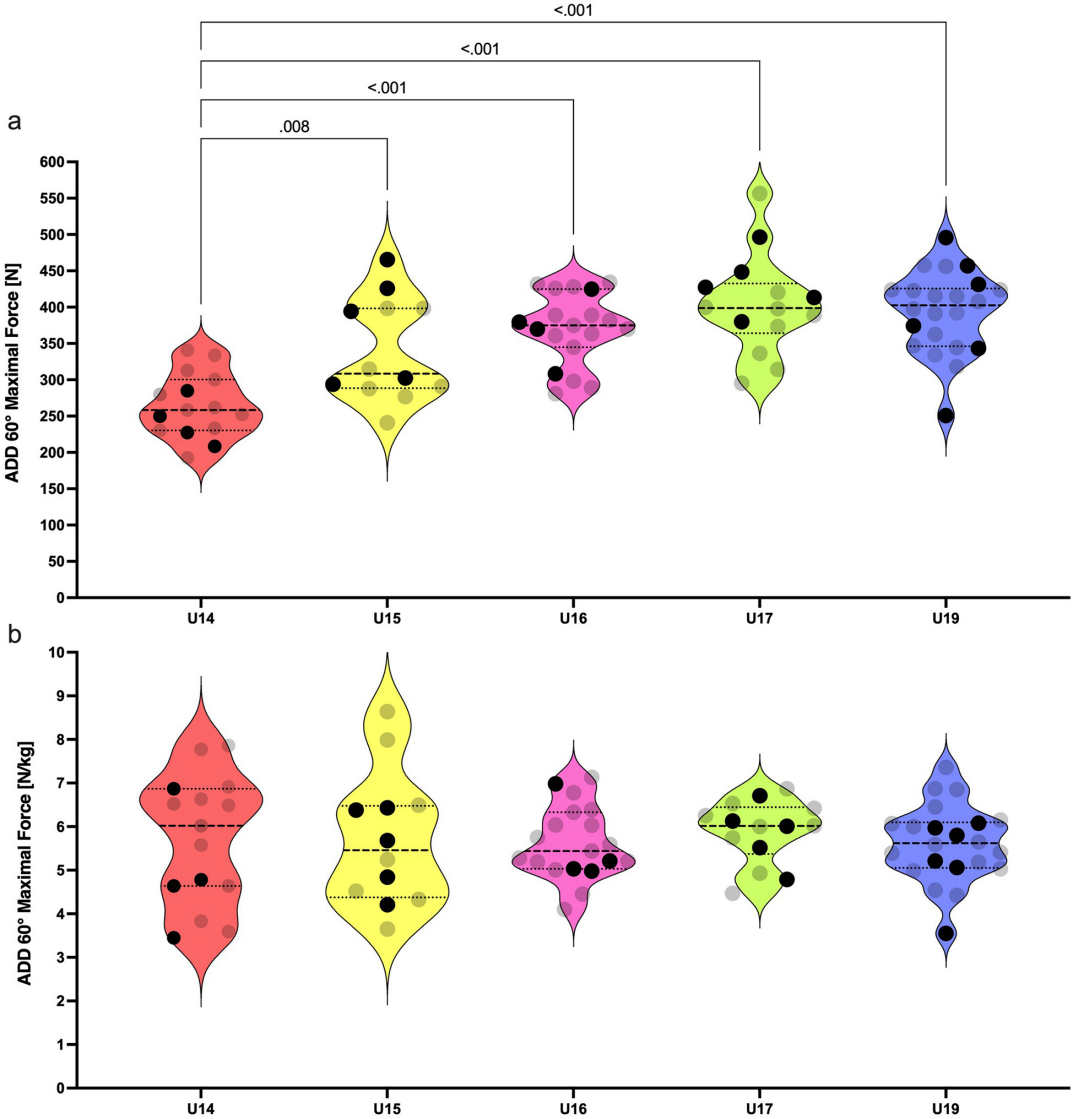
Violin plots for absolute **(a)** and relative **(b)** isometric strength of hip adductors among U14–U19 players. Black dots represent the national team selected players. Only significant differences are highlighted by pairwise comparison (*p* < .05).

Age group influenced maximal force produced in ADD 60° [F _(4, 77)_ = 14.8; *p* = < 0.001; *η*^2^ = 0.43; Figure 3a] with no effect on the relative force in ADD 60° [F _(4, 77)_ = 0.15; *p* = 0.962; *η*^2^ = 0.01; Figure 3b]. The U14 group produced significantly less force than the U15, U16, U17, and U19 groups (ES = 1.28, 2.28, 2.42 and 2.56; respectively). No significant difference, but a large effect size was revealed in the comparison of U15 with U17 and U19 groups (*p* = 0.052 and 0.085; ES = 0.90 and 0.83; respectively).

### 3.2 Comparison of sprinting speed among age groups

**Figure 4:**
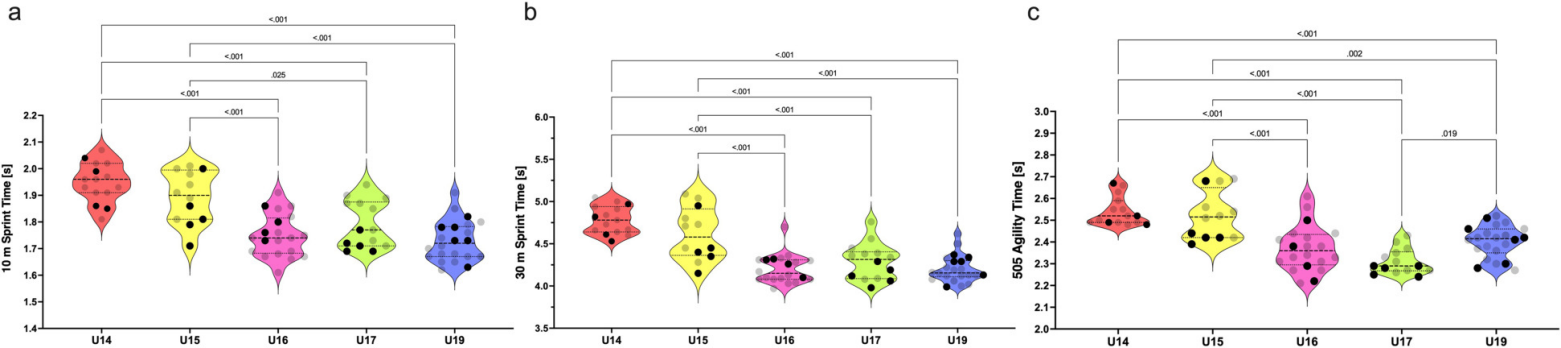
Violin plots for 10 m sprint time **(a)**, 30 m sprint time **(b)**, and change of direction speed time **(c)** among U14–U19 players. Black dots represent the national team selected players. Only significant differences are highlighted by pairwise comparison (*p* < .05).

Age groups had a large effect on the linear speed performance of players in the 10 m sprint test [F _(4, 78)_ = 22,0; *p* = < 0.001; *η*2 = 0.53; Figure 4a], and 30 m sprint test [F _(4, 78)_ = 28.0; *p* = < 0.001; *η*^2^ = 0.59; Figure 4b]. Change of direction speed in the 180° COD test differed significantly among groups [F _(4, 78)_ = 19.7; *p* = < 0.001; *η*^2^ = 0.50; Figure 4c]. Specifically, U14 and U15 groups were significantly slower (*p* = < 0.001 and < 0.025) in the 10 m sprint, 30 m sprint, and 180° COD test than the U16, U17, and U19 groups. ES ranged from 1.95 to 3.68 for U14 differences and 1.07–2.37 for U15 differences.

### 3.3 Comparison of relative isometric strength between selected and non-selected national team players

National team selected players did not produce significantly higher relative force in ISO 30° (–0.08 ± 0.21 *N*/kg; *p* = 0.698, ES = 0.10), ABD 60° (–0.16 ± 0.29 *N*/kg; *p* = 0.586, ES = 0.13), and ADD 60° (–0.33 ± 0.26 *N*/kg; *p* = 0.215, ES = 0.31) compared to their academy peers. Descriptive data for these variables are shown in Table 4.

**Table 4:** Comparison of relative maximal isometric strength between national team (NT) selected (*n* = 24; U14 = 4, U15 = 5, U16 = 4, U17 = 5, U19 = 6) and non-selected (*n* = 59) academy players.

	ISO 30° [*N*/kg]	ABD 60° [*N*/kg]	ADD 60° [*N*/kg]
NT selected	4.90 ± 1.00	5.04 ± 1.14	5.45 ± 0.94
	(4.64–5.33)	(4.52–5.56)	(5.05–5.86)
NT non-selected	4.98 ± 0.79	5.20 ± 1.16	5.78 ± 1.11
	(4.78–5.18)	(4.91–5.49)	(5.50–6.06)
*p* (ES; 95% CI)	0.698	0.586	0.215
	(0.10; –0.59–0.42)	(0.13; –0.64–0.37)	(0.31; –0.84–0.17)

Data are presented as mean ± SD (95% CI). *P*, statistical significance; ES, Cohen's d; 95% CI, 95% confidence interval; ISO 30°, isometric strength test of the knee flexors; ABD 60°, isometric strength test of the hip abductors; ADD 60°, isometric strength test of the hip adductors.

## 4 Discussion

The primary aim of this study was to examine the differences in isometric lower body strength and sprinting speed among five distinct age groups (i.e., U14, U15, U16, U17, and U19) in the elite soccer academy. Results indicate that the isometric strength of knee flexors increased gradually from U14 to U19. The strength of abduction and adduction gradually increased up to the U17 group. Slight decreases in these parameters were observed in the transition from the U17 to the U19 group. Relative force production significantly differentiated only the U15 and U19 groups in the ISO 30° test. The U16 players possess a higher linear and COD speed than the U15 players. Overall, the most sensitive period for neuromuscular demands is their transition from the U15 to the U16 age group. This finding is corroborated by significant differences in isometric adduction strength, linear speed, and COD speed between these age groups. However, the highest differences in isometric strength are visible between U14 and U16 academy soccer players. Surprisingly, U17 players exhibit a higher COD speed than U19 players. Additional findings showed that the relative isometric strength parameters of youth soccer players may not determine their selection for the national team.

### 4.1 Differences in isometric strength among age groups

The isometric strength of knee flexors gradually increased from U14 to U19. However, the strength of abduction and adduction remained comparable among U16–U19 players. We found that the differences in absolute isometric strength are the most noticeable between U14 and U16 academy soccer players. Acceleration in muscular power development takes place near the period of peak height velocity (35). This period is clearly observed between 13.8 and 14.2 years in European boys (36). The U16 players in our study had an average age of 15.1 ± 0.3 years. As is known, mature athletes demonstrate superior performance in strength and power tasks over boys in either early or mid-puberty (37). Probably, the U16 players harness their advantages in higher muscle mass and better motor unit activation than the U14 and some of the U15 players. Additionally, these factors are significantly associated with muscular power (35). This fact can further explain their superior speed performance over U14 and U15 players.A special focus should be placed on the analysis of the isometric adduction strength in U14, U15, and U16 players. The U15 age group had 22% higher adduction strength compared to U14. Even greater differences were evident between U14 and U16, while U15 and U16 did not differentiate significantly. Large effect sizes were calculated from the comparison of U15 with U17 players in all three isometric strength tests (ES = 0.83–1.26). One study showed that 31%–40% of the variance in speed and strength variables of 13–16 years old soccer players can be explained by height and weight interaction, whereas only 6%–10% of the variance accounts for their younger counterparts (38). Firstly, anthropometric parameters must be taken into account when transitioning players between these age groups. Secondly, our perspective accentuates that grouping of players by chronological age overlooks individual variation in neuromuscular performance among young athletes. This issue is apparent in violin plots displaying the relative isometric strength of players (Figures 2–4). Increased intraindividual variance in the U14 and U15 relative strength compared to older groups highlights the importance of biological maturity assessment in these groups. There are several methods for the assessment of maturity in sport. For example, maturity offset can be predicted by the equation including standing height, sitting height, and body mass (39). Other methods, such as the evaluation of ossification on specific bones, can be performed by medical proffesionals (40). These strategies allow players to compete in equal and fair environments. For example, bio-banding is an approach for grouping players according to their biological maturity instead of chronological age (41). This approach can be appropriate before the transition of players into the U16 age group.We found a plateau in isometric strength and speed performance from U16 to U19 age groups. These results are in agreement with Millar (42), who found slight but non-significant increments of the isometric peak force gradually from U16 to U18 and to reserve squad (18.43 ± 1.03 years). It seems that the traditional methods of strength development from the U16 age group are not sufficient to produce significant improvements in maximal isometric strength towards the senior level of soccer. Data from Ishøi et al. (17), showed plateau in isometric strength in transition from U17 to U19 age group which is in accordance with our findings. Interestingly, in our study the U17 group outperformed the U19 group in COD 180° test. Outcome of this test is dependent on relative force production in first 150 and 200 ms of isometric contraction (15). Millar (42) further discovered that eccentric strength measured by the Nordic hamstring exercise differentiated U16 from reserve squad players. The same study demonstrated this pattern in the COD 90° test. COD in soccer includes the decelerative phase of movement where eccentric strength plays an important role in COD performance (43). Older players seem to elicit higher eccentric force, which leads to outperforming younger players in COD tasks. In addition, knee flexors can generate higher peak forces in eccentric contractions than in isometric and concentric contractions (44). These facts give rise to the idea to prefer eccentric over isometric strength testing of players competing in elite youth environments. Overall, these presumptions points towards the fact that the transition of players to the superior age groups among the U16, U17, and U19 teams might not be based on the maximal isometric strength assessment. Isometric muscle contraction is less specific to the dynamic environments of team sports. A poor association among the mechanical outputs of the eccentric and isometric tests can be found in male professional soccer players (45). Further research should elucidate whether significant differences in the eccentric strength of knee flexors among U16 and older age groups would be detectable.

We noticed that maximal isometric strength did not increase significantly from U16 to U19 age group. Neuromuscular performance plateau was previously found in elite spanish youth players in explosive strength, acceleration speed, and COD speed (46). Isolated isometric maximal strength was investigated rarely. Ishøi et al. (17), found plateu in aboslute and relative torque of hamstring muscles from U15 to U19 age group. On the one hand, this plateau is probably caused by the termination of the growth spurts in U16 categories. On the other hand, elite clubs might have an excellent selection of players in the talent identification process, which often emphasizes the physical aspects of performance. Nowadays, these academies are able to prepare outstanding youth players in early adolescence to compete at the senior level. Previous study discovered that elite senior players had lower isometric hamstring torque in comparison to elite senior players (17). The cumulative training load, combined with the specific sprinting load, may be greater for senior players than for younger athletes. This load, which exposes the hamstring muscles to excessive eccentric forces, may lead to a decrease in their maximal strength capacities during the weekly training cycle. In our study, this decrement was slightly present in ABD 60° and ADD 60° tests. The U19 age group produced lower maximal isometric force in abdictuion and adduction than the U17 group (–15 *N*, 0.35 *N*/kg and −10 *N*, −0.17 *N*/kg; respectively). These findings are in accordance with a recent study, which measured almost identical values of isometric adduction strength in long lever exercise (i.e., ∼0° knee flexion) for U17 and U19 soccer players (47). In our case, this result points towards the issue of training and match programming for the U19 group. Demands for the neuromuscular load of the senior team are often replicated for this age group, which might lead to the decrement of some strength parameters compared to younger counterparts.Relative ISO 30° differences were observed exclusively between U15 and U19. Age and maturation increase absolute isometric peak force and impulse in young male soccer players but exert a lesser role in relative strength metrics (48). A high variability in maturation status of U15 players arises from the period of peak height velocity. However, it is not clear why these changes affected the isometric strength of knee flexors but not the abduction or adduction strength. Further research could consider investigating this topic.

### 4.2 Differences in linear and COD speed among age groups

The transition of players from the U15 to U16 age group seems to be most demanding for their level of sprinting speed. A comparison of linear and COD speed pointed to the significantly lower speed performance of the U15 age group (ES = 1.39–1.67). These differences were not found between U14 and U15 players (ES = 0.22–0.69). Previously, the U15 soccer players have been found faster at shorter distances (i.e., the 5 m sprint test) compared to the U17 and adult players (5). This difference could show substantial improvements in relative muscle power throughout the development of youth athletes. However, the evolution of speed performance appears to be inconsistent. Our results indicate that speed performance stabilizes from the U16 to U19 age group. This finding in in accordance with a previous study, which discovered that acceleration speed (<30 m) and COD (180°) speed of elite academy players is stabilized from the U16 to U19 age group. Our findings are in contrast with Malý et al. (49), who found significantly lower 10 m sprint times in U19 compared to U16 and U17 elite soccer players. However, mean differences compared to U17 and U16 groups were of 0.01 s and 0.06 s, respectively. These differences were probably of small effect. We found the U16 players slightly faster in 10 and 30 m sprint speed compared to the U17 group (–0.04 s and −0.09 s, respectively; medium effect). According to Dragijsky et al. (50), it is essential to recognize that performance variances may be ascribed to several reasons, including different training methodologies, the training age of athletes, or their earlier transition into older groups. In summary, practitioners should consider the level of speed when transitioning players from the U15 to U16 age group to secure their readiness for the higher level of performance.

The COD speed extracted from the COD 180° test should not be considered to analyze age-related differences between U17 and U19 players. The group of U17 players was faster than U19 in COD 180°. This difference in favor of U17 age group was not expected to occur. Nearly equal levels of COD speed were previously described for the U17 and U19 elite spanish youth players (46). Age-related differences in the COD test were neither observed in highly trained adolescent soccer players (51). These claims are in contrast to other study, which concluded that U19 players perform better in the COD task than U17 players (52). However, authors used tasks where either soccer-specific skills or reactions to external stimuli were demanded. It seems that the higher ecological validity of the testing methods would bring more insight into the transition of players between the U17 and superior age groups. This underlines the fact that testing of COD speed in reaction to visual stimuli, i.e., reactive agility, can be beneficial for more profound performance analysis of adolescent soccer players (53). In addition, specific modification of strength and conditioning structure within the training periodization should be considered to further improve players towards the senior level of soccer. According to Loturco et al. (54), the volume of technical-tactical workload in each age group should be regulated and supplemented with specific interventions that can be transferred to the game conditions.

Naturally, the U19 age group possesses the fastest linear sprinting speed. This group showed the highest maximal force production in ISO 30° test. Maximal isometric strength and rate of force development measured during an isometric muscle contraction are very closely linked to linear sprinting speed. This fact is documented, for example, in elite sprinters or academy netball players (55, 56). So far, no studies have evaluated the association between sprinting speed and the isolated strength of knee flexors in elite academy soccer players. Based on our findings, the maximum isometric strength of knee flexors may show a similar relationship with the linear speed of U19 players.

Parameters of sprinting speed have implications for the transition of players to the superior age groups. A recent study indicates that an increased sprinting distance during training and competitive matches raises the likelihood of youth players transitioning to the senior squad. A similar trend was observed for high-intensity acceleration and deceleration efforts (57). These assumptions are not fully comparable to the results of our study. Nevertheless, it seems that for the transition of players between age groups, both neuromuscular tests and high-intensity external load variables from soccer matches can be considered.

The speed variables concomitantly with the isometric strength parameters that were previously described showed a minimal age-related difference among U16, U17, and U19 age groups. These findings could be explained by the possible increment in volume of technical-tactical training towards the demanding senior level of elite soccer (58). This increase would naturally decline the volume and general focus of coaches on the speed and neuromuscular development programs in academies. Likely beneficial could be the power and speed training progressively included to training programs of soccer academies from the U16 age groups. Surprisingly, our study discovered that U17 players overcome older peers in COD speed test. Coaches should implement specific training programmes targeting this ability in U18 and U19 age groups. For example, 4-week training programme focusing on the technical aspect of COD movement significantly improves this ability in U18 elite soccer players (59). Practitioners should also consider using other advanced methods in these categories where plateau is more likely to occur.

### 4.3 Comparison of relative isometric strength between national team selected and non-selected players

Isometric strength might be a good indicator to differentiate elites from the non-elite athletes but not the national team (NT) selected players from their academy peers. On the one hand, the elite youth soccer players have greater isometric maximal voluntary force than their non-elite counterparts (26). However, the relative force production in isometric muscle contraction was not higher in the NT selected players as expected. One study compared the isokinetic strength of adult NT players with conventional domestic league players who did not reach the NT level. The greatest differences were found in the concentric strength of knee flexors (60). This is in contradiction with our findings, which did not find NT selected players relatively stronger in either of isometric strength tests. Since the strength and motor performance of adults do not fully match with the adolescents (27), we cannot neglect the importance of muscle strength for the elite players competing in international level.

The distribution of data points for speed and strength parameters of selected NT players does not form any clear patterns (Figures 1–4). However, there are some clues that can be pinpointed in the presented datasets. For example, three of four players from U14 age groups were identically below the median values for relative ISO 30°, ABD 60°, and ADD 60° tests (Figures 1b, 2b, 3b). This finding leads to the probable later maturity offsets of players whose technical-tactical quality overlooks their physical weaknesses. A similar data distribution of NT selected players can be seen in the U15 speed performance data distribution. In this case, four of five NT selected players performed below the median value of this group. Physical underdevelopment of these NT players is pointing towards the importance of technical-tactical and cognitive aspects of soccer performance for the selection of players for the youth NT. However, this suggestion needs to be assessed more profoundly. The main limitation of U14 data is that the selection of players for the NT was based on their selections for the regional team. National teams in Slovakia start from the U15 age group, where about 90–100 players are scouted through the four U14 regional teams. Another shortcoming would be the restricted number of U15 players included in this study (*n* = 12).

A recent study analyzed the speed and explosive strength of female national team soccer players (61). It shows that physical qualities differentiate youth (U17, U19, and U23) and senior national team selected players from non-selected counterparts in 10 and 30 m sprint tests. These differences were analyzed according to the playing positions. We did not find any significant difference comparing pooled groups of players from U14 to U19. Therefore, conducting position-specific analyses of male youth players selected for the NT would be crucial to illuminate the topic of talent identification through neuromuscular assessments. However, the inclusion of NT players into the scientific research is unique and rarely found. It seems to be clear that other factors, such as technical skills or psychological aspects of performance, must be taken into consideration when developing players to compete at the international level. This claim can be supported by the finding of Trajković et al. (53). Authors compared the acceleration speed, COD speed, and reactive agility (i.e., COD in reaction to an external stimulus) among elite, sub-elite, and amateur soccer players. Speed variables were sensitive enough to detect differences between elite/sub-elite and amateur players. However, only the reactive agility test was able to detect elite players from the sub-elite group. This indicates that neuromuscular performance might be a good indicator for talent identification in lower-tier academies, but the inclusion of decision-making and specific movement needs to be used in the assessment methods in elite academies. Consequently, selection of players to the youth national teams includes multidimensional structure of selection criteria. Data from a single country and short-term selections does not offer a full comprehensive view of how players are chosen for the national team in general. As emphasized before, positional differences and different approaches and criteria of various countries need to be considered by further research.

Overall, findings of this research are limited by few issues. The data presented in this study were collected in a single elite soccer academy in Slovakia. These variables could differ in academies using different methodologies in other European countries. Furthermore, data were not collected on the same day of the week and time due to differences in the academy training schedules. However, they were collected at “match day −3” (i.e., >72 hours after the last game) by the identical academy staff and researchers. Secondly, we did not consider the maturity groups within each age group to control for the effects of biological maturity status on testing results. Therefore, we recommend assessing the peak height velocity of players in age groups affected by pubertal biological changes. However, it is important to emphasize that dividing the players according to chronological age is an ecologically valid approach since soccer federation rules are based on this maturation criterion to regulate the competitions. In addition, the longitudinal observation of players’ strength and speed development would lead to further practical applications for youth soccer and strength and conditioning coaches. We believe that further research on this topic would use these suggestions to enhance testing and training strategies based on the developmental needs of young athletes.

## 5 Conclusion

The absolute isometric strength gradually increases from the U14 to U16, while neither isometric strength nor acceleration speed differentiates the U16, U17, and U19 age groups. Higher speed and strength demands are visible in the transition from the U15 to the U16 group. Practitioners may look at the isometric strength and speed parameters for the selection of players to the U16 age group, since these players exhibit the most robust performance differences within the closest inferior and superior groups. To avoid the physical performance plateau, the advanced training methods of speed and strength development should be considered to improve academy players from the U16 age group towards the senior squad. Relatively stronger academy players are not selected more often for the national team. Therefore, other factors, such as technical and tactical skills or cognitive performance, may be more adequate than selection based solely on physical aspects of soccer performance. Selection and evaluation of players quality based on physical parameters alone seems to be insufficient in modern elite soccer. Longitudinal follow-up, comparative analysis across several academies, and assessments of biological maturity are required to further elaborate on the talent identification and equal assessment frameworks for young athletes.

## Data Availability

The datasets presented in this study can be found in online repositories. The names of the repository/repositories and accession number(s) can be found below: https://data.mendeley.com/preview/2z96524k6x?a=9d4be57e-5651-4294-a1a9-22e4c877241d.
